# Tissue Options for Construction of the Neovaginal Canal in Gender-Affirming Vaginoplasty

**DOI:** 10.3390/jcm13102760

**Published:** 2024-05-08

**Authors:** Nicholas C. Bene, Peter C. Ferrin, Jing Xu, Geolani W. Dy, Daniel Dugi, Blair R. Peters

**Affiliations:** 1 Division of Plastic and Reconstructive Surgery, Oregon Health and Science University, Portland, OR 97239, USA; 2 Transgender Health Program, Oregon Health and Science University, Portland, OR 97239, USA; 3 Department of Surgery, Oregon Health and Science University, Portland, OR 97239, USA; 4 Department of Urology, Oregon Health and Science University, Portland, OR 97239, USA

**Keywords:** gender-affirming vaginoplasty, gender-affirming surgery, vaginal reconstruction, penile inversion vaginoplasty, robotic-assisted peritoneal flap vaginoplasty, intestinal vaginoplasty, revision vaginoplasty

## Abstract

Gender-affirming vaginoplasty (GAV) comprises the construction of a vulva and a neovaginal canal. Although technical nuances of vulvar construction vary between surgeons, vulvar construction is always performed using the homologous penile and scrotal tissues to construct the corresponding vulvar structures. Therefore, the main differentiating factor across gender-affirming vaginoplasty techniques is the tissue that is utilized to construct the neovaginal canal. These tissue types vary markedly in their availability, histology, and ease of harvest and have different advantages and disadvantages to their use as neovaginal lining. In this narrative review, the authors provide a comprehensive overview of the tissue types and associated operative approaches used for construction of the neovagina in GAV. Tissue choice is guided by several factors, such as histological similarity to natal vaginal mucosa, tissue availability, lubrication potential, additional donor site morbidity, and the specific goals of each patient. Skin is used to construct the neovagina in most cases with a combination of pedicled penile skin flaps and scrotal and extra-genital skin grafts. However, skin alternatives such as peritoneum and intestine are increasing in use. Peritoneum and intestine are emerging as options for primary vaginoplasty in cases of limited genital skin or revision vaginoplasty procedures. The increasing number of gender-affirming vaginoplasty procedures performed and the changing patient demographics from factors such as pubertal suppression have resulted in rapidly evolving indications for the use of these differing vaginoplasty techniques. This review sheds light on the use of less frequently utilized tissue types described for construction of the neovaginal canal, including mucosal tissues such as urethral and buccal mucosa, the tunica vaginalis, and dermal matrix allografts and xenografts. Although the body of evidence for each vaginoplasty technique is growing, there is a need for large prospective comparison studies of outcomes between these techniques and the tissue types used to line the neovaginal canal to better define indications and limitations.

## 1. Introduction

Vaginoplasty has consistently been one of the most desired gender-affirming surgeries for transfeminine individuals, with some studies describing interest as high as 50% of surveyed subjects [1]. Gender-affirming vaginoplasty (GAV) typically refers to both the construction of a vulva and a neovagina from components of disassembled genital tissue. Although the technical specifics of vulvar construction vary widely, the general principles of replacing “like with like” remains the same across techniques: the penis is disassembled into components which are then used to reconstruct their corresponding vulvar structures or “homologues” [2].

In GAV, a neovaginal canal is created with sufficient width and depth to allow for sexual penetration. The average depth of the neovagina, typically 12–14 cm, is intentionally constructed longer than that of a natal vagina, as tissues used to line the neovaginal canal do not have the same stretch and elasticity. In 1957, Sir Harold Gilles described an early form of penile inversion vaginoplasty (PIV) using pedicled penile skin flaps [3]. A similar technique was independently developed and popularized by the French gynecologist Dr. Georges Burou after the publication of his cohort of over 3000 cases in 1974 [4]. To this day, the PIV remains the most popular technique and other approaches to GAV represent modifications of its original description. Although techniques in vulvar construction have become more standardized, current GAV approaches fall into several categories based on the tissue that is used to construct and line the neovaginal canal. These tissues differ markedly in their availability, histology, and ease of harvest and have different advantages and disadvantages for their use as lining. Regardless of tissue type, a neovagina does not have the same capacity for self-lubrication as a natal vagina does.

The purpose of this article is to provide a review of tissue options that can be used to line the neovaginal canal in cases of GAV.

### 1.1. Skin

Skin is the most common tissue used to line the neovaginal canal [5,6,7] and consists of an outer epidermis composed of stratified squamous epithelium, and a deeper dermis composed of collagen and elastin [8]. Due to its availability in the surgical field, many vaginoplasty surgeons prefer to use all available genital skin for neovaginal lining prior to using alternative tissues.

**Figure 1:**
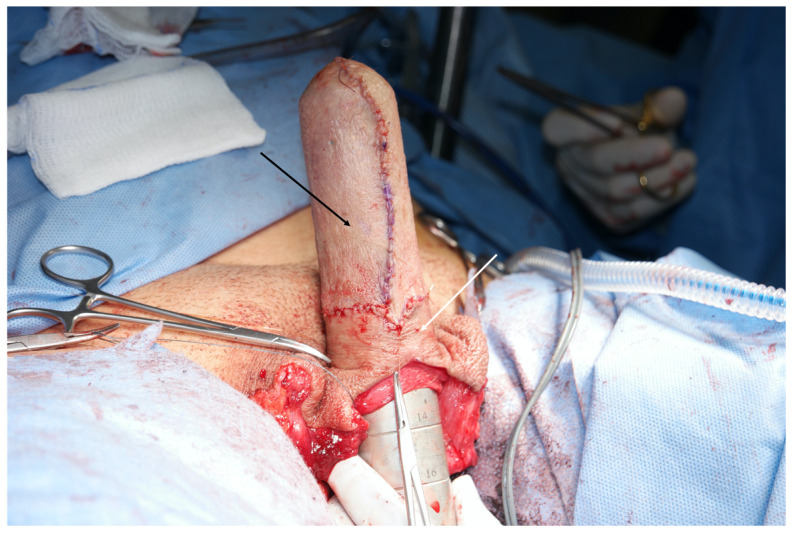
Penile skin (proximal, white arrow) and scrotal skin graft (distal, black arrow) which have been inverted over a vaginal dilator. This tissue will be used to create the lining of the neovaginal canal in the open Penile Inversion Vaginoplasty technique.

Skin can be used in the form of skin grafts or a vascularized penile skin tube, as in the well-described penile inversion vaginoplasty (PIV) [5]. PIV involves degloving the penis, then inverting the proximally-based cylindrical penile skin flap to line the introitus and the neovagina [2,7,9]. Proponents of using penile skin for neovaginal lining note that it is non-hair bearing, sensate, contracts minimally, and minimizes introital scarring. The stretched penile length, which can be determined preoperatively, estimates the amount of penile skin that is available for both vulvar construction and neovaginal lining. Most vaginoplasty patients do not have sufficient penile skin to reach the vaginal introitus and line the entire neovaginal canal. The reasons for this are twofold: first, the average neovaginal depth of 12–14 cm is longer than the general population’s mean penile length of 12.9 cm; [10,11] and second, a significant length of the available penile skin is used to construct the outer vulva and reach the introitus which is several centimeters from the base of the penis. The remainder of the mid and apical neovagina can then be lined by skin grafts (Figure 1).

Full-thickness skin grafts have a higher rate of skin loss, typically have diminished sensation, and undergo secondary contracture compared to vascularized penile skin. Despite these limitations, the rates of vaginal stenosis in PIV techniques with and without skin grafts have been shown to be comparable [12,13]. This may be explained by consistent neovaginal dilation, an important postoperative requirement that may counteract the tendency for graft contracture. Scrotal skin is the most common donor, typically harvested as a full-thickness skin graft. There is usually excess scrotal skin removed as part of the vulvar construction that would otherwise be discarded, and therefore the use of scrotal skin for canal construction does not add morbidity to the patient.

In cases of penoscrotal hypoplasia, there is a global deficiency of genital skin and therefore insufficient penile skin and scrotal skin graft to line the entire neovaginal canal. In this situation, extra-genital skin grafts are needed [2]. Prior orchiectomy has been shown to decrease the amount of scrotal skin available for use as a skin graft at the time of vaginoplasty, thus increasing the chance of needing an extra-genital skin graft three-fold [14]. This is important to highlight during preoperative counseling for patients who are pursuing orchiectomy prior to vaginoplasty. The need for extra-genital skin grafting is also increased in patients who have undergone pubertal suppression, a population that is expanding due to earlier access to gender-affirming hormone therapy [2]. Although extra-genital skin grafts may be harvested as split-thickness or full-thickness grafts, there is a general preference for the latter in order to minimize the degree of secondary contracture and the subsequent risk of neovaginal stenosis [15,16,17]. Preferences for extra-genital skin graft donor sites vary between institutions. Our preferred donor site is the groin, as it allows for scar concealment in a natural skin crease and undergarments. If bilateral groin skin is still insufficient, we will harvest full-thickness skin grafts from the lower abdomen in an abdominoplasty-like incision pattern. Although many other potential skin graft donor sites have been described, we prefer to avoid these due to the visibility of the donor site scar seen with hip and thigh skin grafts or the need for an intra-operative position change seen with buttock and gluteal crease skin grafts [7,15,18,19,20,21].

Whenever skin is used for neovaginal lining, preoperative hair removal of the donor sites is a critical consideration. Intravaginal hair can lead to malodor, discharge, bezoars, and pain with dilation or sexual penetration [22]. Electrolysis is accepted as the gold standard technique for permanent removal of hair but can be both costly and painful [22]. Other modalities, such as laser or intense pulsed light, are often better tolerated but are most effective in treating coarse dark hair in fair-skinned individuals [23]. The time and cost associated with preoperative hair removal can be significant; a mean number of 21 h has been reported for electrolysis-based hair removal in vaginoplasty [24]. For this reason, some surgeons will forgo preoperative hair removal and instead rely on aggressive intraoperative thinning of the hair-bearing scrotal skin with scraping and/or cauterization of follicles. Other surgical teams choose to discard the scrotal skin entirely and harvest skin grafts from non-hair-bearing areas such as the groin or hip, accepting the additional scars as a trade-off to avoid hair removal.

One criticism of skin-only neovaginal lining is the inability of penile skin or skin grafts to produce lubrication. Despite this, most neovaginal canals are noted to be moist at baseline. A recent review demonstrated that neither penoscrotal skin, bowel, nor peritoneum-lined neovaginas provided functional lubrication comparable to a natal vagina, thus concluding that existing evidence does not support choosing one type of neovaginal lining based on lubrication outcomes alone [25].

### 1.2. Peritoneum

The peritoneum is a thin serous membrane composed of visceral and parietal layers. Histologically, each layer is composed of three sublayers: the mesothelium, basal lamina, and submesothelial stroma. The mesothelium is made up of simple squamous cells that house lamellar bodies, which produce lipid-containing secretions for lubrication [25,26].

Utilizing peritoneal lining for vaginal reconstruction was popularized by Davydov in 1974 for cases of congenital vaginal agenesis in the cisgender population [27]. Recently, robotic and laparoscopic approaches that harvest peritoneum have emerged as potential solutions to penoscrotal hypoplasia or limited genital skin in cases of GAV [28,29]. In these approaches, the canal dissection is performed transabdominally in an antegrade direction. This is carried distally until Denonvilliers’ fascia is incised to develop the neovaginal space between the prostate and rectum. Concurrently, a retrograde perineal dissection is carried out beneath the bulbar urethra and through the perineal body until the intra-abdominal and perineal spaces are joined [2]. The intra-abdominal dissection approach affords several advantages: greater attainable canal depth, safer dissection plane (particularly in revision cases), a well-vascularized neovaginal apex, and, in cases of penoscrotal hypoplasia, elimination of the need for extra-genital skin grafts [2,28,30]. However, this technique also introduces additional potential complications including pelvic abscess, injury to intra-abdominal or pelvic structures, and intra-abdominal adhesion formation. Of note, performing peritoneal vaginoplasty as a primary surgical option leaves intestinal vaginoplasty as the main salvage option in the case of complete vaginal stenosis. This is particularly important to highlight during preoperative counseling as patients decide on which technique is best suited to their goals. For this reason, the role of peritoneal flap vaginoplasty as a primary GAV technique is debated. In the authors’ experience in attending transgender health meetings and via direct communication, some centers offer primary GAV using peritoneal flaps to all patients while other centers offer this operation only to patients with insufficient genital skin. Data on the number of centers that offer each option have not been reported in the literature.

**Figure 2:**
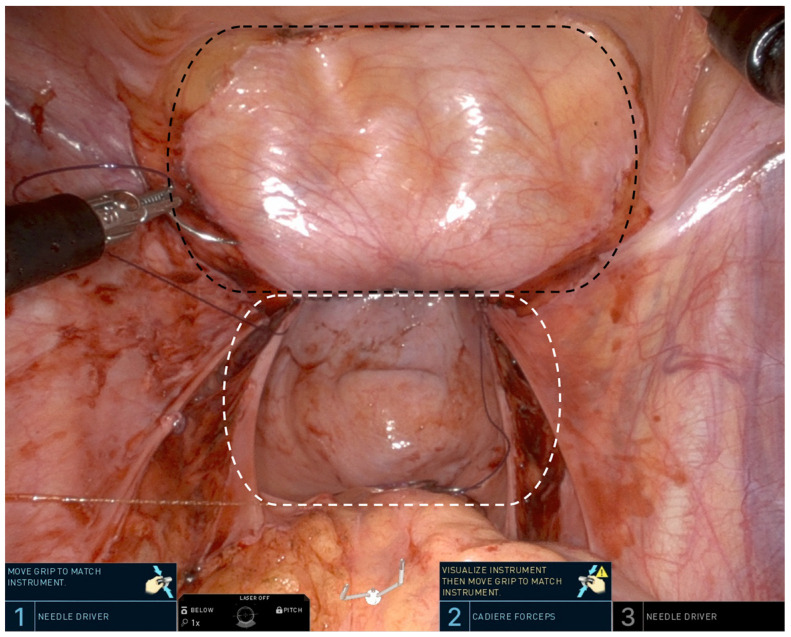
Intra-abdominal view of the peritoneal flaps that will be approximated to create the neovaginal apex. The extent of the anterior peritoneal flap is denoted by the black dashed line and the extent of the posterior peritoneal flap is denoted by the white dashed line.

Due to the lack of data on the effectiveness of peritoneal grafts, most centers use only peritoneal flap techniques, though reports of the use of peritoneal grafts for the reconstruction of congenital vaginal/cervical defects exist [30]. At our center, this involves the harvest of two advancing peritoneal flaps—one from the bladder and another from the rectum—that form the vaginal apex and up to 75% of the neovaginal canal (Figure 2) [2]. These operations are typically performed using the DaVinci Xi or SP platforms due to the superior visualization and access to the deep anterior pelvis afforded by the robotic platform via an antegrade dissection [28,31]. Another described technique, the urachus-peritoneal hinge flap, utilizes a long pedicled flap of peritoneum, urachus, and transversalis fascia from the bladder to the umbilicus. This is folded on itself in a hinge fashion to construct the vaginal apex. Proposed advantages include the lack of a suture line at the apex, inclusion of a substantive urachus, and exclusion of the rectum [32]. Finally, a pedicled peritoneal flap based on the deep inferior epigastric artery has been described that is first externalized through a groin incision, then delivered into the neovagina extra-peritoneally through a separate perineal incision [33]. No studies have directly compared these differing peritoneal flap techniques.

The peritoneum—a semi-permeable membrane—normally secretes a small amount of transudative fluid. Whether this basal fluid provides clinically significant lubrication long-term remains controversial. In our experience, although the peritoneum-lined neovagina can be naturally moist, it does not produce sufficient fluid in the setting of erogenous stimulation and arousal, and exogenous lubrication is needed for dilation or sexual penetration [2]. The peritoneum-lined canal in cisgender patients with vaginal agenesis has been shown to undergo metaplastic changes to squamous epithelialization at about six months to one year postoperatively [2,34]. Although the lining can grossly appear similar to natal vaginal mucosa, the characteristic rugae formed by natal submucosal smooth muscle fibers are absent [35]. No similar histologic studies have been performed in transgender patients following peritoneum-based vaginoplasty techniques to confirm whether a similar process of metaplasia is seen.

**Figure 3:**
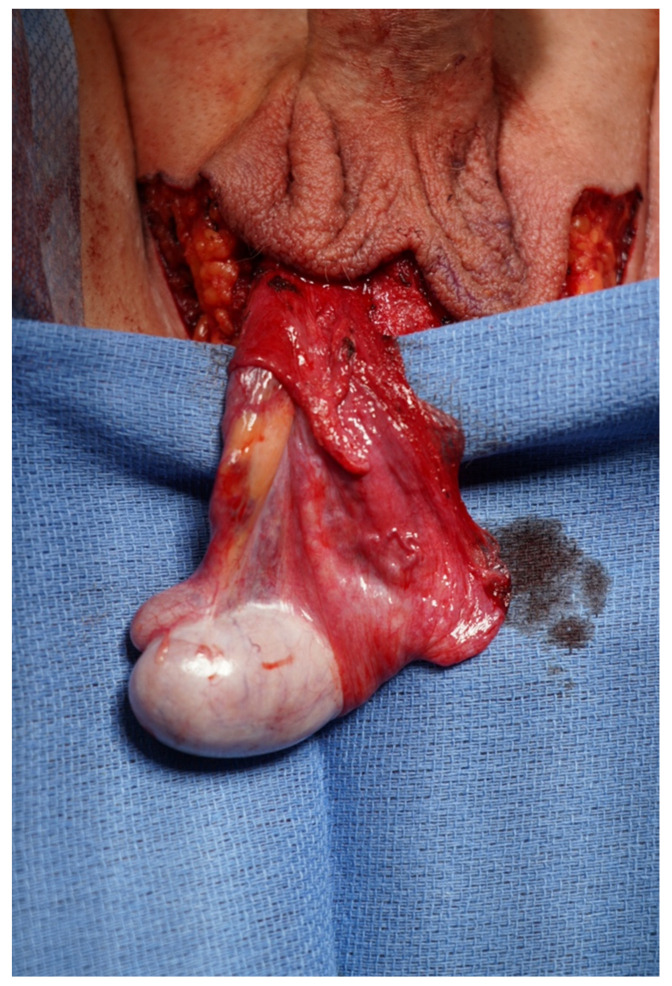
Tunica vaginalis covering the testis.

The use of tunica vaginalis to supplement neovaginal lining has been reported by several vaginoplasty centers without data on outcomes [36,37]. The tunica vaginalis derives from the abdominal peritoneum, continuing through the inguinal canal to surround the testis and epididymis [38]. The tunica vaginalis consists of two layers, a visceral tunica (tunica vaginalis propria) and a parietal tunica (tunica vaginalis communis). The visceral tunica adheres firmly to the tunica albuginea and covers the testis. However, the parietal tunica is continuous with the parietal peritoneum of the abdomen and forms a sac that lines the scrotal cavity. In cases without prior orchiectomy, the parietal tunica can be harvested and used as a graft to provide partial lining of the neovaginal canal. Proponents of this technique describe the use of tunica vaginalis as graft for additional neovaginal lining in cases of penoscrotal hypoplasia where skin is deficient, and there is a desire to avoid the use of abdominal surgery to harvest peritoneum or intestine. Criticisms include the small amount of tunica vaginalis available and the very thin tissue of questionable durability and quality for grafting (Figure 3). In our practice, we do not use tunica vaginalis grafts as we prefer the reliability of vascularized peritoneal flaps from the abdomen.

### 1.3. Intestine

The sigmoid colon is the most common intestinal segment used in GAV. The colon is comprised of four layers: the mucosa, submucosa, muscularis, and serosa. Histologically, the colonic mucosa is a simple columnar epithelium with tubular glands—the Crypts of Lieberkuhn—that contain mucin-producing goblet cells [39]. The ileum, less commonly used, is composed of four similar layers with one distinctive feature—Peyer’s patches. These are clusters of lymphatic tissue within the lamina propria that facilitate the immune response to pathogens.

Intestinal vaginoplasty was first reported by Baldin in 1907 in cisgender patients with vaginal agenesis [6]. Presently, this technique is most commonly used as a salvage or secondary vaginoplasty option after a prior failed GAV [39]. Although uncommon, some centers offer intestinal vaginoplasty as a primary procedure for patients with limited genital skin [40]. Intraoperatively, the vascular pedicle of a bowel segment is first identified, then the intestine is mobilized and anastomosed to the neovaginal introitus. The bowel segment is typically suture fixated to the sacral promontory to avoid mucosal prolapse [40]. The remaining bowel ends are then anastomosed to restore continuity [6]. Proponents of this technique note that the bowel provides adequate depth and has minimal tendency to shrink or contract. This also eliminates the need for extra-genital skin grafts, particularly in cases of penoscrotal hypoplasia [39].

Sigmoid colon vaginoplasty is thought to provide a large lumen, trauma-resistant thick walls, and a possible decreased need for dilation [41]. However, Bouman et al. noted that, in the literature, there is still a 0–56% incidence of introital stenosis following sigmoid vaginoplasty [40,42]. There are also several additional complications specific to sigmoid colon vaginoplasty including the possibility of developing diversion colitis [42,43], inflammatory bowel disease [44], or malignancy within the neovaginal segment [45]. Diversion colitis can be diagnosed by neovaginoscopy and presents as colonic inflammation caused by the lack of luminal nutrients, particularly short-chain fatty acids [39]. This can be treated with topical short-chain fatty acids, 5-aminosalicylic acid, or steroids [40]. The ileum, on the other hand, is used less frequently. Wu et al. described harvesting a 15–20 cm length of ileum located 50–70 cm from the ileocecal valve. They cite advantages of this approach compared to the colon, including less mucus production and discharge and no risk of diversion colitis [43,44,45].

Due to the morbidity of bowel harvest and the additional risk of gastrointestinal complications, as well as the issue of malodorous discharge and potential for diversion colitis, intestinal vaginoplasty is not typically offered as a primary vaginoplasty procedure. At our institution, if patients have insufficient genital skin, then we will offer primary vaginoplasty with extra-genital skin graft harvest or the use of peritoneal flaps for neovaginal lining. In our practice, consideration for use of intestinal vaginoplasty is limited to cases of complete obliteration of the neovaginal canal following peritoneal flap vaginoplasty.

### 1.4. Mucosa

The mucosa of the urethra has been reported for use in neovaginal lining as both a flap and a graft. Histologically, urethral mucosa is lined by stratified columnar epithelium. The underlying lamina propria contains mucus-secreting cells—the Littre glands—that provide lubrication and protect the urethra from urine and ejaculate.

Perovic et al. have described their technique using a vascularized urethral flap to line the inside of the penoscrotal skin tube [46]. In cases of insufficient genital skin, they report that the urethral flap can reach beyond the proximal end of the penoscrotal skin tube. Other centers have used a similar approach by joining the urethral tissue to the penoscrotal skin tube as a vascularized flap or graft [47]. The listed advantage of utilizing urethral mucosa is that there is usually excess length available, which would otherwise be discarded. There have also been reports of basal mucous secretions from this type of neovaginal lining [48]; however, long-term outcome studies are sparse and a recent review demonstrated that urethral mucosa-lined neovaginas did not provide functional lubrication comparable to a natal vagina [25].

Autologous buccal mucosa was first reported for use in genital surgery in hypospadias repair by Humby in 1941 [49]. Later, in the 1980s, buccal mucosa became a popular option for urethral reconstruction [50]. Proponents of this tissue for use in genital reconstruction have cited that histologically, compared to skin grafts, the buccal mucosa has a thicker epithelium and thinner lamina propria, which theoretically promotes revascularization [51]. While there have not been published studies on the use of buccal mucosal grafts in GAV, the literature discusses their use in the cisgender vaginal agenesis population. Reported advantages include adequate match in color and texture, lack of hair, and reported mucus production [20]. Postoperatively, the neovaginal lining has been histologically confirmed to be mucosal [52] and stratified squamous epithelium [49]. Furthermore, analysis of the neovaginal discharge revealed this to be mucus [52]. However, the major drawback of this approach is the size of graft available for harvest—reportedly up to about 8 cm × 3 cm—providing only a small fraction of the surface area of tissue needed for total neovaginal lining (140 cm^2^ or more), in addition to the morbidity of intra-oral tissue harvest. Due to the availability of local genital tissues, the use of buccal mucosa is not currently practiced in the field of GAV and the use of urethral mucosa remains relatively limited.

### 1.5. Allograft and Xenograft

Allografts and xenografts have been described for use in neovaginal lining. Rodriguez et al. [53] report using Nile tilapia fish skin: in this technique, the fish skin xenograft is tubularized and sutured to the penile skin tube, akin to a scrotal graft in PIV. Reported histologic analysis of the lining 6 months post-operatively revealed a hyperplastic epithelial lining similar to that of a natal vagina [54]. AlloDerm (LifeCell, Branchburg, NJ, USA), an allograft, has also been utilized in GAV. This is an acellular dermal matrix that is created by removing the epidermal and cellular components of human cadaveric skin while preserving the basement membrane and collagen skeleton of the extracellular matrix. This framework allows for epithelialization within 3–6 weeks while remaining non-immunogenic. Parker et al. described using AlloDerm in revision peritoneal flap vaginoplasty cases when there is insufficient peritoneal flap length to reach the remnant vaginal canal [55]. The allograft was used in lieu of their prior approach of utilizing extra-genital skin grafts to bridge this gap. The authors used two pieces of 4 × 7 cm extra-thin AlloDerm (thickness 0.38–76 mm) to create a tube around a vaginal dilator. This tube is then interposed and sutured to the edges of the peritoneal flap and remnant canal. Although these reported techniques both eliminate additional donor site morbidity, experience with their use is limited, and long-term outcomes, including tissue incorporation and lubrication, are unclear. The additional cost of tissue processing should also be considered ($30/cm^2^ for AlloDerm) [55]. Most surgical teams do not offer the use of xenografts due to skepticism of their viability and the abundant availability of autologous tissue options. In one series, 22% of patients were demonstrated postoperatively to have areas of excess AlloDerm that did not epithelialize and required excision. This area was noted to be at the junction of the allograft and peritoneal flaps [55]. The use of allografts is generally reserved for complex revision cases where more common lining options such as skin and peritoneum are insufficient.

### 1.6. Revision Surgery

Regardless of technique, GAV requires strict adherence to dilation schedules postoperatively to maintain neovaginal canal width and depth. This is especially important for the first months to years after surgery, though for most patients can be a lifelong commitment. Post-operative complications or difficulty with adherence to dilation can lead to neovaginal stenosis. Revision vaginoplasty is most often performed in the setting of significant neovaginal stenosis or, in more severe cases, complete canal obliteration.

In cases of revision vaginoplasty, the genital skin has already been utilized at the primary operation, necessitating lining the neovaginal canal with alternative tissue. This is often achieved with either extra-genital skin grafts [15], intestine [56,57], or increasingly, with the use of peritoneum [28,29], depending on the original operative technique employed. Revision vaginoplasty is technically challenging given difficult surgical access from the perineum, a paucity of additional tissue to mobilize for secondary flap creation, and obliterated tissue planes adjacent to critical structures (e.g., bladder, urethra, and rectum). Prior to revision surgery, systematic analysis of the cause of stenosis must be carried out and any modifiable risk factors must be addressed to avoid restenosis, a problem for which a surgical fix is extremely difficult or even impossible.

Revision following PIV with skin is often performed using skin grafts, intestine, or peritoneal flaps. A study directly comparing outcomes after revision intestinal vaginoplasty versus revision skin graft vaginoplasty found that the intestinal technique was associated with a lower rate of rectal injury (10% vs. 19%, although not statistically significant), as well as significantly longer surgical time and lower rates of restenosis [56]. In the case of a revision vaginoplasty after an initial PIV, peritoneal flap vaginoplasty is becoming the technique of choice due to higher rates of morbid complications such as rectal injury with secondary perineal dissection. This technique offers several advantages that make it particularly well suited for revision surgery: the robotic antegrade approach is safer than a high-risk repeat retrograde perineal dissection due to a scarred field and allows for mobilization of fresh tissue planes from within the abdomen [28].

Unfortunately, revision after a primary peritoneal flap GAV is typically limited to intestinal vaginoplasty. This is one criticism of performing peritoneal flap vaginoplasty as a primary operation in patients with sufficient genital skin for a traditional PIV. However, as access to gender-affirming care continues to improve and patients can access pubertal suppression, the utilization of primary peritoneal flap-based vaginoplasty may continue to increase to avoid the need for extra-genital skin grafts. Concurrently, the use of intestinal vaginoplasty for revision following stenosis or obliteration in these cases may increase to reflect this trend.

In our practice, patients with sufficient genital skin are encouraged to pursue traditional PIV with the use of skin grafts as needed to line the neovaginal canal. If a revision vaginoplasty is needed in the future, then a peritoneal flap vaginoplasty is offered. On the other hand, patients with insufficient skin are offered peritoneal flap vaginoplasty as a primary operation. If a revision is needed after peritoneal flap vaginoplasty, then intestinal vaginoplasty is considered. For patients who have had a prior vulvoplasty and wish to undergo a subsequent vaginoplasty, we offer either open vaginoplasty with the use of extra-genital skin grafts or peritoneal flap vaginoplasty depending on patient preference and comorbidities.

## 2. Conclusions

Gender-affirming vaginoplasty comprises a range of techniques, differing mainly in the tissue used to line the neovaginal canal (Table 1). Skin is used to construct the neovagina in the majority of cases with a combination of penile skin flaps, scrotal, and extra-genital skin grafts. However, skin alternatives such as peritoneum, intestine, mucosa, and allograft/xenografts are increasing in use. Peritoneum and intestine are emerging as options for primary vaginoplasty in cases of limited genital skin or revision vaginoplasty procedures. The increasing number of gender-affirming vaginoplasty procedures performed and the changing patient demographics from variables such as pubertal suppression have resulted in rapidly evolving indications for the use of these differing vaginoplasty techniques. Although the body of evidence for each vaginoplasty technique is growing, there is a need for large prospective comparison studies of outcomes between these techniques and the tissue types used to line the neovaginal canal to better define indications and limitations.

## Figures and Tables

**Table 1:** Advantages and disadvantages of each tissue type used for neovaginal canal lining. XGSG = extra-genital skin graft; IBD = inflammatory bowel disease.

Tissue Type	Advantage	Disadvantage	Key References
Skin graft	Available within genital surgical field (scrotal skin) Avoids intra-abdominal surgery	Need for hair removal Tendency for secondary contracture Higher risk of skin loss Insensate Additional donor site (XGSG)	Hage et al. (2007) [4] Buncamper et al. (2016) [7] Sineath et al. (2022) [14]
Vascularized skin	Reliable perfusion Sensate Minimal contracture and scarring	Limited availability (stretched penile length)	
Peritoneum	Large surface area available Well-vascularized tissue (flaps) Avoids XGSG	Requires intra-abdominal approach and related risks Often requires two surgeon approach	Jacoby et al. (2019) [29] Dy et al. (2021) [28,31]
Bowel	Provides adequate depth Potential for less contracture Avoids XGSG Possible decreased frequency of dilation	Risk of mucosal prolapse Risks related to intra-abdominal surgery and bowel anastomosis Risk of diversion colitis, IBD, or malignancy within neovaginal segment Malodor and excess mucus discharge Often requires two surgeon approach	Bouman et al. (2016) [40] Van der Sluis et al. (2016, 2019) [5,56]
Mucosa	Use of tissue that would otherwise be discarded (urethra) Non-hair bearing Adequate match in color and texture	Sparse availability of tissue Possible addition of intra-oral donor site (buccal)	Macedo et al. (2023) [20] Perovic et al. (2000) [46] Gentile et al. (2020) [48]
Allograft and Xenograft	Avoids donor sites	High cost Unclear viability Minimal data on outcomes	Rodriguez et al. (2020) [53] Dias et al. (2020) [54] Parker et al. (2023) [55]
